# Renin-angiotensin system: The underlying mechanisms and promising therapeutical target for depression and anxiety

**DOI:** 10.3389/fimmu.2022.1053136

**Published:** 2023-01-24

**Authors:** Sizhu Gong, Fang Deng

**Affiliations:** Department of Neurology, First Affiliated Hospital of Jilin University, Changchun, China

**Keywords:** deprssion, anxiety, renin-angiotensin system, angiotensin II, neuroinflammation

## Abstract

Emotional disorders, including depression and anxiety, contribute considerably to morbidity across the world. Depression is a serious condition and is projected to be the top contributor to the global burden of disease by 2030. The role of the renin-angiotensin system (RAS) in hypertension and emotional disorders is well established. Evidence points to an association between elevated RAS activity and depression and anxiety, partly through the induction of neuroinflammation, stress, and oxidative stress. Therefore, blocking the RAS provides a theoretical basis for future treatment of anxiety and depression. The evidence for the positive effects of RAS blockers on depression and anxiety is reviewed, aiming to provide a promising target for novel anxiolytic and antidepressant medications and/or for improving the efficacy of currently available medications used for the treatment of anxiety and depression, which independent of blood pressure management.

## 1 Introduction

Emotional disorders, including depression and anxiety, contribute considerably to morbidity across the world (1). Anxiety disorders are the most common class of disorders listed in the Diagnostic and Statistical Manual of Mental Disorders, Fifth Edition (DSM-V), which are complex interactions between biological, psychological, temperamental, and environmental factors (2). As a group, anxiety disorders represent a heterogeneous group of illnesses that are characterized by excessive fear and anxiety, hypervigilance, and related behavioral disturbances (3). Furthermore, anxiety is one of the most common comorbid disorders with major depressive disorder (MDD) (4, 5). A large psychiatric cohort study has reported that depression preceded anxiety in 18% of such comorbid cases, while in 57% of the cases anxiety preceded depression (5). Comorbid anxiety and many core depression symptoms may be caused by hyperactivity of the hypothalamic-pituitary-adrenal (HPA) axis combined with amygdala dysfunction (4). The amygdala is a key element of anxiety circuitry and produces behavioral responses associated with fear and anxiety by integrating information from sensory inputs in the cortex and thalamus (6). Similarly, some neuroimaging studies have reported enhanced amygdala glucose metabolism and activation in patients with depression (7), and that depression-associated anxiety is accompanied by an increase in amygdala volume (8). First-degree relatives of individuals with anxiety have an increased risk of developing anxiety disorders or depression (9). Importantly, however, only 40–70% of patients with depression respond to pharmacological treatment and therapies often have a delayed onset (10), and anxiety associated with depression often leads to reduced responses and decreased compliance with pharmacotherapy (6). Consequently, emotional disorders are a serious public health issue, and identifying novel targets for their treatment is worthy of further attention.

The role of the renin-angiotensin system (RAS) in hypertension and emotional disorders is well established. Evidence points to an association between elevated RAS activity and depression and anxiety, partly through the induction of neuroinflammation, stress, and oxidative stress (11). Importantly, blocking RAS can have anti-inflammatory and anti-oxidative stress effects, providing a theoretical basis for future treatment of anxiety and depression. Captopril and enalapril (angiotensin-converting enzyme inhibitors; ACEIs) may rapidly improve depressive moods in hypertensive patients (12). This has sparked significant interest in RAS targets. The evidence for the positive effects of RAS blockers on depression and anxiety is reviewed here to evaluate a promising target for novel anxiolytic and antidepressant medications. Furthermore, this knowledge may aid the improvement of the efficacy of currently available medications used for the treatment of anxiety and depression, which are independent of blood pressure management.

## 2 Overview of RAS

RAS-blockers or RAS inhibitors are classes of medications that block the renin-angiotensin axis, primarily inhibiting angiotensin (Ang) II activity. Examples include ACEIs and selective Ang II type 1 receptor blockers (ARBs) (13). The most common ACEIs include captopril, enalapril, lisinopril, perindopril, ramipril, and imidapril. The most common ARBs include losartan, irbesartan, candesartan, telmisartan, and valsartan. As effective first-line antihypertensive medications, ACEIs and ARBs have been shown to minimize the risk of cardiovascular and renal events as well as mortality (14). These classes of drugs target Ang II, however, differences in their mechanisms of action impact their effects on other pathways and receptors, which may have therapeutic implications. For example, ACEIs inhibit RAS activation by preventing the conversion of Ang I to Ang II, resulting in reduced activation of both Ang II type 1 (AT1/AT1) receptors and Ang II type 2 (AT2/AT2) receptors (15). Moreover, ACEIs prevent the degradation of Ang-(1–7) by angiotensin-converting enzyme (ACE), thereby the level increased due to a build-up caused by the lack of degradation of Ang-(1–7) (15). Additionally, ACEIs block the degradation of bradykinin, leading to activation of the β-2 receptor and promotion of nitric oxide (NO) release with vasodilatory and tissue-protective effects (13). One study showed that ACEIs rapidly ameliorate depressive behaviors *via* the bradykinin-dependent activation of the target of the rapamycin complex (10). Unlike ACEIs, ARBs block RAS by antagonizing the binding of Ang II to the AT1 receptor and activating the AT2 receptors, thus producing insufficient Ang II to elevate Ang-(1–7) level (16). High levels of Ang-(1–7) reduce anxiety and depression behaviors, providing positive benefits (see below). RAS blockers shift the balance to increase circulating levels of Ang-(1–7), this may contribute to shunt the ACE/Ang II/AT1 pathway toward the ACE2/Ang-(1–7)/MasR pathway providing beneficial effects on mood disorders (17).

Although RAS is widely acknowledged as a cardiovascular circulation hormonal system, it is found in a variety of organs, including the brain. The RAS is composed of two pathways that are mutually antagonistic that maintain the balance through angiotensin-converting enzyme 2 (ACE2): the classical pathway angiotensin-converting enzyme/angiotensin II/angiotensin II type 1 receptor (ACE/Ang II/AT_1_R) and the non-classical pathway angiotensin-converting enzyme 2/angiotensin- (1–7)/Mas receptor (ACE2/Ang-(1–7)/MasR).

### 2.1 Classical pathway: ACE/Ang II/AT_1_R

The classical pathway contains renin, angiotensin (Ang) II, angiotensin-converting enzyme (ACE), angiotensin II type 1 receptor (AT_1_R/AT1R), and angiotensin II type 2 receptor (AT_2_R/AT2R). Renin is an aspartyl protease typically produced in the juxtaglomerular cells of the kidney, and it cleaves angiotensinogen (ATN, an inactive peptide formed and secreted by the liver) to produce angiotensin I (Ang I). Ang I has few physiological effects and produces Ang II as a substrate for ACE. Ang II exerts several physiological effects: constriction of blood vessels, stimulation of aldosterone secretion, and release of catecholamines. Ang II acts by binding to the AT_1_R and AT_2_R. When Ang II activates the AT_1_ receptor, it causes neurotoxicity, such as vasoconstriction, pro-inflammatory, apoptotic, and anti-diuresis. Furthermore, increased circulating levels of Ang II disrupt blood-brain barrier (BBB) integrity, allowing circulating Ang II to access the brain parenchyma and trigger the AT_1_R directly, producing oxidative stress and brain inflammation (18). AT_2_R is activated by Ang II and may counterbalance AT_1_R neurotoxic effects and determine a neuroprotective role in RAS activation, such as vasodilation, diuresis, anti-fibrosis, antihypertensive, and cognitive improvement. AT_2_R activation is important in blunting the negative effects of AT_1_R, such as neuroinflammation and oxidative stress (19). However, evidence shows that AT_1_ receptors predominate in adult tissues and AT_2_ receptors predominated in the developing brain (20).

### 2.2 Non-classical pathway: ACE2/Ang-(1–7)/MasR

The non-classical axis is neuroprotective and composes angiotensin-converting enzyme 2/angiotensin-(1–7)/Mas receptor axis (ACE2/Ang-(1–7)/MasR). The non-classical axis exerts neuroprotective effects, such as promoting the release of NO and promoting anti-inflammatory, anti-fibrotic, and vasodilatation effects. In the brain, all components of the ACE2/Ang-(1–7)/MasR axis are expressed. ACE2 correlates with AT_1_R and Ang II levels and ACE2 overexpression results in the downregulation of AT_1_R and increases the expression of AT_2_R and MasR. As a homologous enzyme of ACE, ACE2 is found in the hippocampus and cerebral cortex that cleaves Ang II to produce Ang-(1–7), which activates the Mas receptor and produces an inverse regulation of the ACE/Ang II/AT1 pathway (21, 22). Ang-(1–7) generated in the rat hippocampus has been reported (23). As for the Mas receptor, it was a G protein-coupled receptor specific for Ang-(1–7), which is expressed in brains and other different organs, including the hippocampus, amygdala, and cortex (21).

## 3 The relationship between RAS and depression/anxiety

**Figure 1:**
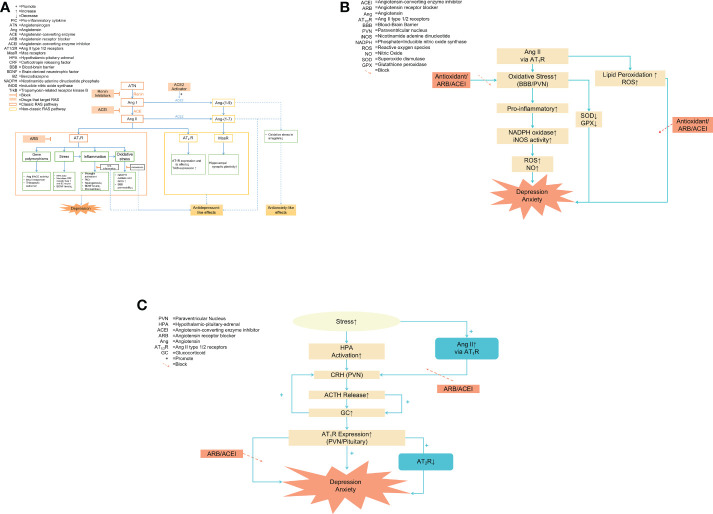
**(A)** The overview of RAS and the role of the RAS in the pathophysiology of anxiety and depression. Notes: The blue dashed line pointed to the positive effects, such as antidepressant-like and anxiolytic effects. Two key regulatory pathways: the classical axis ACE/Ang II/AT1 receptor pathway and the non-classical pathway ACE2/Ang-(1–7)/Mas receptor pathway. Under physiological conditions, the two pathways regulated each other and maintained a dynamic balance. Ang II aggravated oxidative stress and inflammation response by activating AT1R to upregulate the ACE/Ang II/AT1R pathway, promoting the development of emotional disorders. Ang II activated non-classical pathways by activating AT2R and MasR, producing antidepressant-like and anxiolytic effects. Thus, the beneficial effects of RAS blockers may be due to inhibiting oxidative stress and inflammation by directly targeting Ang II and its AT1 receptor. Other potential targets of anxiolytic drugs include renin, ACE2, AT2 receptors, and Mas receptors. **(B)** RAS and oxidative stress in depression and anxiety Notes: The pro-inflammatory effects of Ang II were largely mediated by increased oxidative stress. During inflammation, Ang II activated AT_1_R to promote the oxidative stress process by increasing NADPH oxidase and iNOS activity in the BBB and PVN, resulting in an accumulation of ROS and NO and ultimately aggravating emotional disorders. Antioxidant enzyme activity was decreased during oxidative stress, which was prevented by RAS blockers and antioxidants. Furthermore, Ang II directly increased ROS generation and triggered lipid peroxidation, inhibited by RAS inhibitors and antioxidants. **(C)** HPA and RAS. Notes: The type of stress caused HPA axis activation and increased the downstream hormones such as CRH, ACTH, and GC, eventually promoting anxiety and depression. Stress increased Ang II levels, which in turn raised the expression of CRH mRNA, induced the release of ACTH and GC, and enhanced the stimulatory effects of CRH through AT1R located in the pituitary and PVN. The absence of AT2 receptor transcription enhanced the AT1 receptor expression in brain areas and was involved in regulating the HPA axis, which was associated with anxiety and depression. Pretreatment with RAS blockers attenuated neuroendocrine responses, preventing the development of stress-induced anxiety/depression-like behaviors. **(C)** RAS and oxidative stress in depression and anxiety. Notes: The pro-inflammatory effects of Ang II were largely mediated by increased oxidative stress. During inflammation, Ang II activated AT_1_R to promote the oxidative stress process by increasing NADPH oxidase and iNOS activity in the BBB and PVN, resulting in an accumulation of ROS and NO and ultimately aggravating emotional disorders. Antioxidant enzyme activity was decreased during oxidative stress, which was prevented by RAS blockers and antioxidants. Furthermore, Ang II directly increased ROS generation and triggered lipid peroxidation, inhibited by RAS inhibitors and antioxidants.

In addition to the systemic RAS, all the components of the RAS independently exist in the brain involving the pathophysiology of depression and anxiety. Hyperactivation of the ACE/Ang II/AT1R classical pathway accelerate the disease process *via* activated AT1R, while AT2R plays a protective role (24). We will discuss the evidence and mechanisms of RAS involvement in depression and anxiety in this part (**Figure 1A**) (**Table 1**).

**Table 1:** The relationship between RAS components and depression/anxiety.

RASComponent	Compounds	Species	Mode of Administration	Measure Method	Main Findings	Reference
**ATN**		Low ATN TGR			Anxiety/depression-like behaviors↑	(25)
**Ang II**		UMS rats	21 days	OFT	Anxiety-like behaviors↑ Cognition↑	(26)
		Adult C57 mice	14 days		Anxiety-like behaviors↑ Synaptic plasticity↓	(27)
		Adult male C57BL/6	21 days	TST FST	Depressive−like behaviors↑ HPA axis↑	(28)
		Male SD rats	Microinjected into hippocampal CA1 0.1µg	EPM	Anxiolytic-like	(29)
**AT_1_R**		(AT1A−/−) mice		ETM	Anxiolytic Fear responses↓	(30)
		(AT1A−/−) mice in PVN		EPM OFT	Anxiolytic	(31)
**AT_2_R**	Agonist Novokinin	Male Wistar rats with T1DM	ICV	EPM TMRA	Anxiolytic Cognition↑ Spatial memory↑	(32)
	Antagonist PD123319	Male Wistar rats	Microinjected into MeA (100 nL/side)	EPM FST	Anxiety-like behaviors↑	(33)
	Antagonist PD123319	Male Wistar rats and female C57BL/6 mice		FST	Anxiety-like behaviors↑	(34)
		AT2R-deficient mice		EPM OFT	Anxiety-like behaviors↑	(35)
**ACE2**		Male mice overexpressing ACE2		EPM	Anxiolytic	(36)
	Activator diminazen aceturate	C57BL/6 mice		EPM	Anxiolytic	(36)
Ang-(1–7)		Adult male TGR (ASrAOGEN)	1µmol/µL	EPM FST	Anxiolytic Anti-depressant	(25)
		Adult male Wistar rats		EPM	Anxiolytic	(37)
		TGR rats (mRen2)27	1µL	EPM FST NSF	Anxiolytic Antidepressant	(38)
		Adult male SD rats	0.5μg in 0.5μL	OFT EPM	Anxiolytic	(39)
**Mas**		Mas-deficient rats		EPM	Anxiety-like behaviors↑	(40)
	Antagonist A-779	Male Wistar rats	Microinjected into MeA (100 nL/side)	EPM FST	Anxiety-like behaviors↑	(33)
	A-779	C57BL/6 mice		EPM	Anxiety-like behaviors↑	(36)

ATN, angiotensinogen; UMS, unpredictable mild stress; OFT, open field test; FST, forced swim test; TST, tail suspension test; EPM, elevated-plus-maze; ICV, intracerebroventricular; AT_2_R, AT2 receptors; AT_1_R, AT1 receptors; AT1aR, AT1a receptors; PVN, paraventricular nucleus; ↓, means decreased; ↑, means increased; TGR(ASrAOGEN), transgenic rats with low brain angiotensinogen; TGR, transgenic rat; (AT1A−/−), AT1A receptor knockout mice; ETM, elevated T-maze; TMRA, T-maze rewarded alternation test; T1DM, type 1 diabetes mellitus; MeA, medial amygdaloid nucleus; IP/i.p., intraperitoneally; LDB, light-dark box; NIH, novelty-induced hypophagia; NSF, novelty suppressed feeding; VCT, vogel conflict test; MasR, Mas receptors; ACEI, angiotensin-converting enzyme inhibitors; ARB, angiotensin II receptor blockers.

### 3.1 ACE/Ang II/AT1R and depression/anxiety

Angiotensinogen is the glycoprotein precursor of angiotensin II. Voigt et al. were the first time to describe transgenic rats with low brain angiotensinogen behavioral phenotype as characterized by increased anxiety-related behaviors (41). Subsequently, studies showed low angiotensinogen concentration in the brain leads to anxiety-like behaviors accompanied by a depression-like state (25).

Ang II was previously discovered as a pro-hypertensive factor present in areas of the brain associated with cardiovascular and has recently been found to be associated with motor activity, anxiety, learning, and memory (20). Additionally, increased Ang II level is significantly associated with depression, anxiety, hyperactivity of the HPA axis, and stress (28, 42). For instance, treatment with Ang II for 14 consecutive days had significant anxious-like behaviors and bidirectional synaptic plasticity impairment, and increase expression of GABA_A_Rα1 (γ-aminobutyric acid A receptor) (27). Administered Ang II for 3 weeks induced cognitive impairment and anxiety-like behaviors as shown by spending less time in the four center squares in the open field tests (OFT) (26). Telmisartan and imipramine reversed chronic Ang II infusion-induced behavioral changes, including changes in TST and forced swimming test (FST) (28). Losartan microinjects into the hippocampus CA1 region showed an anxiolytic-like effect in bilateral olfactory bulbectomy rats (OBX, rat model of depression), indicating the involvement of Ang II in the pathogenesis of anxiety by activating AT_1_R (42). Whereas microinjections Ang II (0.1,0.5,1.0µg) into the CA1 hippocampal area, at a dose of 0.1µg shows some anxiolytic effects manifested as an increasing number of entries into the open arms in the elevated plus maze (EPM) (29). The results are inconsistent with previous studies, but there may be anxiolytic and anxiety effects of Ang II in a dose-related U-shaped manner.

Furthermore, hyperactivation of AT_1_a receptors is associated with promoting anxiety-like behaviors in the brain (31). Deletion of AT_1_a receptors (AT_1_a−/−) from the paraventricular nucleus (PVN) attenuated anxiety-like behaviors in rodents as manifested by increased time spent in the open arms of the EPM (31). Further, (AT_1_a−/−)mice reduced flight behavior in the elevated T-maze test (a model of anxiety and panic) and diminished fear responses despite threat levels (30).

Chronically infused intracerebroventricular (i.c.v.) AT_2_ receptors agonist evokes anxiolytic-like effects (32). Treatment with selective AT_2_ receptor antagonist PD123319 decreased the open arms exploration in EPM and changed the pattern of swimming during the FST (33). AT_2_ receptor-deficient mice increased anxiety-like behaviors, which can be reversed by captopril, and show no depression-like behaviors compared to wild-type mice, providing a theoretical basis for ACEIs for the treatment of emotional disorders (35). Recently, it was reported that the modulatory role of the AT_2_ receptor in the development of depressive-like behavior (43). Administration of AT_2_R antagonist PD123319 into the prefrontal cortex reversed the antidepressant effect of losartan (34), indicating AT_2_R has positive effects on depression and anxiety.

### 3.2 ACE2/Ang-(1–7)/MasR and antidepressant/anxiolytic effect

Walther was the first to discover that the ACE2/Ang-(1–7)/MasR pathway is associated with the development of anxiety, demonstrating that upregulating ACE2 significantly improved anxiety-like behaviors (40). ACE2 is essential for maintaining the balance between ACE2/Ang-(1–7)/Mas receptor and the ACE/Ang II/AT_1_R pathway. The main function of ACE2 is to inhibit ACE activity by decreasing Ang II bioavailability and increasing Ang-(1–7) levels. Thus, the overexpression of ACE2 not only is related to the upregulation of AT_2_R and Mas receptors but also to the downregulation of AT_1_R and ACE (44). Elevated ACE2 activity decreases anxiety-like behaviors and inhibits stress-induced activation of the HPA axis in male mice (45). However, in female mice, increasing ACE2 expression only produces anxiolysis without reversing HPA axis activity (45). Consistent with the effect, central administration of diminazen aceturate to mice, an ACE2 activator reduces anxiety-like behaviors in EPM (36).

Ang-(1–7) is associated with reduced depressive and anxious behaviors as a selective non-competitive antagonist of Ang II at type 1 Ang II receptors (46). Overexpression of circulation Ang-(1–7) produced anxiolytic-like effects have been found in transgenic rats (37), which were manifested by increasing the percentage of time spent and frequency of entries in the open arms and decreasing stretching in closed arms of the EPM (38, 47). In addition, overexpression of Ang-(1–7) reverses the increase in heart rate associated with emotional stress and demonstrates less anxious behaviors in transgenic rats (48). Low angiotensinogen levels in the brain lead to anxiety-like behaviors and depression-like behaviors, while intracerebroventricular administration of Ang-(1–7), selective serotonin reuptake inhibitor fluoxetine, enalapril (ACEI) attenuated behavioral changes in transgenic hypertensive rats, as shown by spending a lower percentage of time in the open arms of EPM and decreasing immobility time in FST (25, 38).

Centrally injecting the MasR antagonist reverses the ACE2 and Ang-(1–7)-induced anxiolytic effects, indicating the anxiolytic effects of ACE2/Ang-(1–7)/MasR pathway due to activate Mas receptors (36, 47). Pre-treatment with A779 (a selective Mas receptor antagonist) enhances the anxiety-like effects (38), showing decreases open arms exploration in the EPM and changes the pattern of swimming during the forced swim test (33). MasR-deficient mice influence hippocampal synaptic plasticity and exhibit increased anxiety behaviors in EPM (40). Taken together, the upregulation of the ACE/Ang II/AT1R pathway accelerates the process of the emotional disorder and the non-classical axis ACE2/Ang-(1–7)/MasR has neuroprotective effects on emotional disorders.

### 3.3 RAS blockers-induced mood-elevating effects

ACEIs and ARBs have been shown to have protective and potential therapeutic benefits in mood disorders (49)(**Table 2**). Saavedra et al. proposed that Ang II expression is associated with mood disorders and that reducing brain Ang II levels might decrease anxiety and depression in animal models (57). For example, treatment with captopril had significant antidepressant activity as shown by the forced swim-induced behavioral despair (immobility) test in mice. In addition, it reversed escape deficits in the learned helplessness model (55, 58). Studies in rodents injected with losartan systemically and locally to the anterior prefrontal cortex and medial amygdaloid nucleus have shown antidepressant effects as evidenced by decreased immobility time in the FST (33, 34).

**Table 2:** Anxiolytic/anti-depressant effect of RAS blockers.

RAS Component	Compounds	Species	Mode of Administration	Measure Method	Main Findings	Reference
**ARB**	Losartan	Male RHR (Wistar strain albino rats)	5, 10 mg/kg q.d. orally	OFT EPM SIT	Anxiolytic	(50)
		Male SHR	15 mg/kg/d for 2 months oral gavage	NORT OFT	Cognition↑ Neuroplasticity↑	(51)
		Male Wistar rats	10 mg/kg, i.p.	OFT	Anxiolytic Cognition↑	(43)
		Male Wistar rats	Microinjected into MeA (100 nL/side)	FST	Anti-depressant	(33)
		Male Wistar rats; female C57BL6/j mice	10, 30, 45 mg/kg, i.p.	FST	Anti-depressant Cognition↑	(34)
	Candesartan	Male SHR	1 mg/kg/d for 4 weeks intragastric administration	OFT NORT MWM	Anxiolytic Cognition↑	(52)
	Valsartan	Male Wistar rats	10 mg/kg orally	OFT EPM CAR Chimney test	Anxiolytic Cognition↑	(53)
	Telmisartan	C57 mice with high-fat diet	8 mg/kg q.d. Oral gavage	OFT OPRT Barnes maze	Anxiolytic CBF↑	(54)
		Adult male C57BL/6		TST FST	Anti-depressant	(28)
**AECI**	Captopril	AT2R-deficient mice	0.1, 1.0 mg/kg, i.p.	EPM OFT	Anxiolytic	(35)
		Male Wistar A.F. rats	4, 8, 16, 32 mg/kg/day, i.p.	Learned Helplessness Paradigm	Anti-depressant	(55)
	Enalapril	Male RHR (Wistar strain albino rats)	4 mg/kg q.d. orally	OFT EPM SIT	Anxiolytic	(50)
	Perindopril	Male SD rats	0.1, 1.0 mg/kg/d	Water maze EPM	Anxiolytic Cognition↑ Spatial memory↑	(56)
		Male SHR	1 mg/kg/d for 4 weeks intragastric administration	OFT NORT MWM	Anxiolytic Cognition↑	(52)

FST, forced swim test; OFT, open field test; OBX, bilateral olfactory bulbectomy rats (a rat model of depression); SHR, spontaneously hypertensive rats; NORT, Novel-Object Recognition Test; MWM, Morris Water Maze Test; SD rats, Sprague–Dawley rats; i.c.v., Intracerebroventricular; CAR, Conditioned Avoidance Responses; PFC, prefrontal cortex; RHR, renal hypertensive rats; SIT, social interaction test; CBF, cerebral blood flow; OPRT, object place recognition test, TST, tail suspension test; FST, forced swimming test; MeA, medial amygdaloid nucleus i.p., intraperitoneally; HBP, hypertensive patients.

Chronic administration of the ACEI perindopril has anxiolytic effects on rats (56). Chronic cerebral hypoperfusion induces ACE/Ang II/AT1R overexpression in the hippocampus and causes anxiety. Candesartan and perindopril attenuate anxiety-like behavior and improve memory impairment by downregulating the ACE/Ang II/AT1R pathway and upregulating the ACE2/Ang-(1–7)/MasR pathway in the hippocampus (52). Ang II-induced rats spent significantly less time in the open arms of the elevated plus maze (EPM), this effect was abolished by the administration of valsartan and losartan (53, 59). Losartan effectively attenuated hyperactivity and anxiogenic behaviors in mice as seen in the EPM, social-interaction tests, and open field tests (OFT) (51) (50). Moreover, telmisartan treatment prevented diet-induced anxiety-like behaviors in behavioral tests (54).

## 4 Potential mechanisms of RAS blockers-induced antidepressant/anxiolytic effects

### 4.1 RAS-related gene and development of depression

The main RAS-related gene polymorphisms are found in the angiotensinogen gene, ACE, and the angiotensin 1 receptor gene, which have alleles associated with high levels of Ang II, high ACE activity, and elevated Ang II response, respectively. The ACE insertion/deletion (I/D) polymorphism determines functional variations of the ACE gene that significantly influence ACE plasma concentrations, which account for 30–40% of the variation in plasma ACE levels (60). Baghai et al. investigated the genetic association between 35 single-nucleotide polymorphisms (SNPs) and an I/D polymorphism in the ACE gene. They reported that carrying the T-allele is correlated with higher ACE serum activity, in which the highest ACE activities are found in patients homozygous for the T-allele, and the lowest is noted in patients homozygous for the A-allele (61). This research indicates that enhanced ACE activity is associated with depressive symptoms and increased susceptibility to affective disorders (61). In addition, a study based on the Iranian population showed that high serum ACE activity is associated with the pathogenesis of depression (62). The GG genotype of the A2350G polymorphism is associated with MDD and exhibits significantly higher serum ACE activity than AA or AG. Furthermore, certain variants of the ACE gene, such as the D allele, are more frequently noted in patients with affective disorders and associated with a higher risk of affective disorders (63). The D allele may be associated with the severity of depression in DD genotypes carriers of ACE I/D polymorphism (64).

In fact, in individuals with depression, the ACE I/D polymorphism is significantly associated with HPA axis hyperactivity. Patients with depression carrying the D/D variant of the ACE gene show considerably greater activation of the HPA axis (65). Cortisol secretion is increased in patients carrying homozygous T-alleles, showing higher HPA axis activity (61). In addition, the I/D polymorphism of the ACE gene is associated with both late-life depression and cortisol secretion (66).

Polymorphisms of the RAS-related gene variants have also been associated with a higher risk of depression (67). Saab et al. collected buccal cells from 132 patients with major depression and their first-degree relatives (case controls) in Lebanon (67). Their study showed that the angiotensin receptor type 1 (A1166C) CC genotype is more common in patients with depression, indicating that the CC genotype is significantly associated with depression (p=0.036) (67). A population-based cohort study found the ACE gene (rs1799752) is associated with the incidence of major depression in older individuals in followed up for over 12 years (68). Moreover, variation in the angiotensin II type 1 receptor has been linked with depression diagnosis and frontotemporal brain volumes (69). Two haplotype-tagging SNPs, rs10935724 (p=0.0487) and rs12721331(p=0.0082) showed statistically significant changes in frequency between diagnostic cohorts (69).

### 4.2 RAS-related gene and depression therapeutic outcome

Clinically, the ACE I/D polymorphism seems to influence the therapeutic outcome in patients with depression, including the onset of action of antidepressant pharmacotherapies and the responses to selective serotonin reuptake inhibitors (SSRIs) (70), which plays an important role in the individualized treatment of depression. To investigate the impact of ACE2 gene variants on the antidepressant efficacy of SSRIs, a randomized, controlled trial was completed, involving 200 patients with newly diagnosed depression who underwent fluoxetine or sertraline for 6 weeks, along with ACE2 allele genotyping (71). The result showed that the patients with GA and AA genotypes respond significantly better to sertraline and confirm the role of G8790A in response to some SSRIs (71). Elevated levels of substance P are associated with mood symptoms, such as depression (72) and treatment with substance P receptor antagonists has antidepressant properties (73). DD allele carriers possessing higher ACE activity can promote the degradation of substance P, which may be related to having a positive impact on antidepressant treatment efficacy (63, 72). Another study conducted a survey among 313 patients with depression receiving various antidepressant treatments and found patients with the D/D and I/D genotypes have shorter hospitalization durations and better treatment outcomes than those with the I/I genotype (74). Surprisingly, when the 313 patients were classified by sex, the ACE I/D polymorphism only influences the therapeutic outcome in women with major depression, not men. This may be due to the sex-dependent influence of the ACE I/D polymorphism on therapeutic outcomes in antidepressant therapies through the influence of gonadal hormones (74). Based on this study, the D allele has a beneficial effect on the onset of therapy for depression and can be used as a predictor of faster onset of different antidepressant treatments, but the I-allele seemed to have a delayed effect on therapy (74). Another study enrolled 273 patients with MDD who received various antidepressant treatments and assessed the severity of depression with the Hamilton Depression Scale-17 (HAMD) before and after 4 weeks of therapy (75). That study revealed that patients carrying the D allele respond better to antidepressant treatment than those carrying other genotypes (75). More than 70% of AT1 CC homozygotes have a 50% reduction in the HAMD-17 scale within 4 weeks of antidepressant treatment, implying that patients with a haplotype combining the CC and DD/ID genotypes respond better to treatment than those with a single allele (75). Although the therapeutic outcome in various genotypes is related to pharmaceutical variety, the findings demonstrate that the ACE gene may generate varied antidepressant effects.

### 4.3 RAS blockers inhibit neuroinflammation

Inflammation is triggered by cellular damage caused by infection or injury. The term “neuroinflammation” refers to an immune-related process that occurs within the brain and spinal cord as a result of harm induced by infection, psychological or physical stress, or indirectly as a result of infection emerging in the periphery (76). Subsequently, innate immune cells in the brain (microglia, astrocytes, and oligodendroglia) are activated in response to inflammatory stimuli including the production of cytokines, chemokines, and secondary inflammatory mediators such as prostaglandins (77).

The brain lacks T- and B-cells that are involved in cellular and humoral immunity but contain innate immune cells, including macrophages and dendritic cells (78). Macrophages in the brain referred to as microglia, are the primary immune cells and contain surface membrane receptors recognizing neurotransmitters and hormones (79). Microglia respond to local and systemic inflammatory stimuli by producing pro-inflammatory cytokines (PIC), including interleukin-1α and β (IL-1α and IL-1β), tumor necrosis factor α (TNF-α), and interleukin-6 (IL-6) (79). In addition, microglia produce the anti-inflammatory cytokines IL-10 and transforming growth factor (TGF)-β. Activated microglia also trigger a chain reaction between chemokines, prostaglandins, and NO. In addition to the direct promotion of brain inflammation, chemokines such as CCR2 promote the recruitment of peripheral immune cells into the brain, thereby increasing the effects of inflammation (80).

Appropriate central inflammatory responses are essential to protect the brain from infection and restore homeostasis; however, prolonged inflammation is harmful. Long-term neuroinflammation leads to the activation of peripheral macrophages and central microglia and nerve dysfunction (76). Moreover, excessive responses may lead to decreased levels of brain-derived neurotrophic factors chronic inflammation, and neuronal damage. Sustained activation of the immune response increases inflammation and nitro-oxidative stress, ultimately leading to changes in monoamine levels that increase the risk of many neurological and psychiatric disorders (81). Such a procedure most likely occurs in chronic psychiatric disorders such as depression (82).

In recent years it has become apparent that inflammation is associated with psychiatric disorders. For example, depression is characterized by a chronic low-grade inflammatory state, increased levels of peripheral inflammatory cytokines, and microglial activation (83–85). Clinically, high levels of inflammatory markers are associated with the development of depression (83). In the general population, an elevated C-reactive protein (CRP) level is linked to a higher risk of developing depression (86). Elevated levels of inflammatory cytokines have been observed in both peripheral and cerebrospinal fluid in patients with depression (87). Particularly, elevated levels of circulating pro-inflammatory mediators have been found in patients with treatment-refractory depression (TRD), including TNF-α, IL-6, IL-1β, CRP, and macrophage inflammatory protein-1 (88).

Consequently, it has been hypothesized that medications that suppress levels of pro-inflammatory cytokines might also contribute to treating depression (87, 89). Some antidepressants with known anti-inflammatory effects have been shown to reduce the level of IL-6 and IL-1β in patients with MDD (88, 90). The use of anti-cytokine and anti-tumor necrosis factor drugs (e.g. infliximab, etanercept, and adalimumab) has been associated with significant improvements in depressive symptoms (91). LPS promotes the activation of microglia and induces depressive-like symptoms, while treatment with anti-inflammatory medication alleviates depressive symptoms (92, 93).

In addition to the regulation of blood pressure, the RAS is also an important regulator of the inflammatory states in the nervous system (94). Excessively elevated Ang II levels enhance plasma cytokine levels such as IL-6, interferon-γ (IFN-γ), TNF-α, and IL-1β (95). IL-6 levels increase the most after Ang II infusion, and plasma IFN-γ levels also increases significantly (96). Cytokine expression is controlled at the transcriptional level by pro-inflammatory transcription factors, such as nuclear factor kappa B (NF-κB) and activator protein-1 (AP-1) (97). Ang II induces the differentiation of immune cells and promotes the production of cytokines through NF-κB and/or AP-1, initiating an inflammatory cascade that leads to microglial activation (98). These findings suggest that the RAS has an intimate and complex regulatory role in the immune system.

ARBs have been proven to effectively inhibit inflammation by reducing gene expression of brain pro-inflammatory cytokines (99)(**Table 3**). Ang II facilitates the production of IL-1β and NO. This effect is reversed by losartan, which inhibits NF-κB and AP-1 (110). Administration of candesartan reduces brain AT1R synthesis and inhibits LPS-induced acute brain inflammation throughout the inflammatory cascade, including decreased production and release to the circulation of centrally acting pro-inflammatory cytokines; reduction of brain pro-inflammatory cytokines, cytokine, and prostanoid receptors; and reduced microglial activation (105, 106). Pretreatment with candesartan (1 mg/kg/d, for 3 d before the LPS treatment) lessens LPS-induced ACTH and corticosterone release and reduces gene expression of cyclooxygenase-2 (COX-2), IL-6, and TNF-α (105). Moreover, candesartan prevents the synthesis and release of the pro-inflammatory hormone aldosterone (108). In the pituitary, candesartan decreases the expression of the genes for IL-6, iNOS, and COX-2 (109). It also lessens the release of inflammatory markers such as TNF-, IL-1, and IL-6 in the circulation (109). AT1 receptor blockades are demonstrated to provide superior neuroprotective properties to ACE inhibition (107). In the rat model of neuroinflammation, candesartan (1 nM) inhibits LPS-induced neuroinflammation more effectively even at lower dosages and increases AT2R and anti-inflammatory IL-10 expression than perindopril (1 μM) (107). Systemic administration of telmisartan directly ameliorates the IL-1β-induced neuronal inflammatory response and inhibits oxidative stress (102). Administration of telmisartan attenuates chronic intermittent hypoxia (CIH)-induced neuronal apoptosis and decreases levels of CD45 (leukocyte common antigen), CRP, and IL-6 in the hippocampus and circulation through inhibiting inflammatory response (103). Intranasal administration of telmisartan (1 mg/kg; two months) significantly reduces glial activation in the brain and ameliorates the synthesis of NO, iNOS, TNF-α, as well as IL1-β (104).

**Table 3:** RAS blockers inhibit inflammation.

RAS Component	Compounds	Species	Mode of Administration	Measure Method	Main Findings	Reference
**AT1R**		AT1aR knockout mice		EPM	Anxiolytic Neuroinflammation↓	(31)
**ACE2**		Male SD rats	Bilateral microinjected ACE2 into PVN		Anxiolytic PIC↓	(44)
**ARB**	Irbesartan	Swiss albino mice of UCMS	40mg/kg i.p./p.o.	MFST TST OFT	Antidepressant 5-HT levels↑	(100)
	Telmisartan	Wistar rats with DM	0.05mg/kg, p.o. for 21days	FST OFT EPM	Antidepressant NO↓, IL-6↓, IL-1β↓	(101)
		SK-N-SH human neuroblasts Primary rat cortical neurons	10 ng/ml		Neuronal inflammatory response to IL-1β↓ COX-2 PGE2↓ JNK/c-Jun pathway↓	(102)
		Male SD rats	10 mg/kg for 8 weeks		CD45, IL-6, CRP↓	(103)
		5XFAD mice Primary neonatal rat glial cells	1 mg/kg/day intranasal for 2 months		TNF-α, IL1-β↓ iNOS↓ Aβ burden and CD11b↓	(104)
	Candesartan	WH rats and SHR	1 mg/kg per day for 14 days	EPM	Anxiolytic PIC↓ Microglia activation↓	(105)
		Male SD rats	1 mg/kg oral gavage for 2 weeks	EPM FST NSFT	Anxiolytic Antidepressant IL-1β, IL-6, Cox2↓, iNOS↓, IL-10↑	(106)
		Male SD rats	0.1 mg/kg Orally for 5 days		Astroglia, microglial, STAT3 activation↓ NFкB↓ TNF-α↓ PP2A activation↓ IL-10↑	(107)
		Male Wistar Hanover rats	1 mg/kg/d, s.c. for 3 days		PIC↓ COX-2, IL-6↓ LIF, IκB-α↓	(108) (109)
	Losartan	Microglial cells	10^-5^m		IL-1↓ NF-κB ↓ AP-1 activation↓	(110)
		Wistar rats	ICV 50 μg		NF-κB↑ AP-1↑	(98)
		Wistar rats of DM	20 mg/kg for 2 weeks	FST OFT	Antidepressant Neuroinflammation↓	(111)
		Male LACA mice of CRS	20 mg/kg for 30 days	FST	Antidepressant Insulin levels↑ Locomotor activity↑	(112)
**ACEI**	Lisinopril	Wistar rats	ICV 50 μg		NF-κB↑ AP-1↑	(98)
	Enalapril Ramipril	Wistar rats of DM	(40mg/bwkg/d) (10μg/bwkg/d) for 2 weeks	FST OFT	Antidepressant IL-1a mRNA↓ IL-6 mRNA↓ TNF-α mRNA↓	(113)
	Ramipril	Male LACA mice subjected to CRS	10, 20mg/kg for 30days	FST	Antidepressant Locomotor activity↑	(112)
		Male SD rats	1 μM Orally for 5 days		Astroglia, microglia, STAT3 activation↓ NFкB↓ TNF-α↓ AT2R expression↑	(107)
	Captopril	Male SD rats	0.5 mg/ml for 2 weeks		TNF-α↓ PIC↓	(114)
		MRL/lpr lupus-prone mouse model	5 mg/kg every other day i.p. for 2 weeks	Rotarod Test FST EPM	Antidepressant 5-HT levels↑ IFNα levels↓ Microglial activation↓	(115)

IL-1β, interleukin-1β; IL-6, interleukin-6; NO, nitric oxide; SD, Sprague-Dawley; DM, diabetes mellitus; MR, mineralocorticoid-receptor; TNF-α, tumor necrosis factor-α; IL-1a, interleukin-1a; i.c.v., intracerebroventricular; AP-1, activator protein-1; PIC, pro-inflammatory cytokine; i.p., intraperitoneal; p.o., oral route; CRS, Chronic restraint stress; IFNα, interferon-α; MFST, Modified forced swim test; TST, tail suspension test; OFT, open-field test; UCMS, unpredictable mild stress, WH rats, Wistar Hannover rats; MWM, Morris water maze; PA, passive avoidance; MBT, Marble burying task; NSFT, Novelty-Suppressed Feeding Test; Cox-2, cyclooxygenase-2, NOS, Nitric oxide synthase, LIF, leukemia inhibitory factor; iNOS, inducible nitric oxidase synthase; MIF migration inhibitory factor; NFκB Nuclear factor-kappa B; pSTAT3, Phosphorylated signal transducer and activator of transcription 3; PP2A, Protein phosphatase-2A; PGE2, prostaglandin E2; JNK, c-Jun N-terminal kinase; NOS, Nitric oxide synthase; CIH, chronic intermittent hypoxia; CD45, leukocyte common antigen; CRP, C-reactive protein; 5XFAD, five familial Alzheimer’s disease transgenic mouse; CD11b expression, a marker for microglia; SPT, sucrose preference test; BDNF-TrkB-CREP, brain-derived neurotrophic factor-tropomyosin receptor kinase B-cyclic adenosine monophosphate response element-binding protein.

Consequently, it is hypothesized that medications with anti-inflammatory effects might also have antidepressant potential. Losartan and ramipril can reverse depression-like behaviors in restraint-stressed mice and insulin resistance through anti-inflammatory mechanisms (112). Administration of irbesartan reduces the level of inflammatory mediators and reverses Ang II-induced depressive-like behaviors as manifested by decreased immobility times in the modified forced swim test (MFST) and the TST (100). Pretreatment with losartan significantly improves FST performance and prevents LPS-induced anhedonia and anxiety-like behaviors in addition to preventing LPS-induced higher levels of the pro-inflammatory cytokine (TNF, IL-1, and IL-6) (99, 116). A model of diabetes-associated depression rats exhibited depression-like behavior, which can be therapeutically reversed by losartan (20 mg/kg) *via* altering diabetes-induced neuroinflammatory responses (111). Telmisartan effectively reduces the concentration of pro-inflammatory mediators, including NO, IL-6, and IL-1β, in depressed rats with diabetes (101). Moreover, in a rat model of post-traumatic stress disorder (PTSD), treatment with captopril decreases pro-inflammatory cytokines levels and inhibits microglial activation in the hypothalamus (114). More importantly, the anxiolytic/antidepressant effects of RAS blockers may be mediated by their anti-inflammatory effects, providing new treatment directions.

### 4.4 RAS blockers inhibit oxidative stress

Oxidative stress occurs when there is an imbalance between the production of reactive oxygen species (ROS) and endogenous antioxidant enzymes. Antioxidant enzymes, such as catalase (CAT), superoxide dismutase (SOD), and glutathione peroxidase (GPX), maintain low levels of ROS *in vivo*. Excessive ROS generation and exhaustion of anti-oxidative defense-produced pro-inflammatory mediators results in damage to vital macromolecules and induces cellular apoptosis (117). Another major consequence of ROS-derived damage is lipid peroxidation. The brain is particularly susceptible to damage from reactive oxygen species because of its elevated oxygen consumption and lower levels of endogenous antioxidant enzymes (118).

Many studies have highlighted associations between oxidative stress and the development of affective disorders, and that antioxidants can improve symptoms of emotional disorders (119). Increased levels of oxidative stress markers, pro-inflammatory cytokines, and lipid peroxidation are observed in patients with anxiety and depression (120). Depression patients have significantly higher levels of F8-isoprostanes and lower GPX activity, two markers of oxidative stress, compared to healthy controls (121). Moreover, antioxidant treatment improves diabetes-induced depressive-like behaviors, increases levels of antioxidant enzymes CAT and SOD in brain tissue, and reduces oxidative stress in the hippocampus (118). Additionally, the administration of antioxidants shows an anxiolytic effect (122, 123). F2-isoprostanes and oxidized glutathione are positively associated with total Hamilton Anxiety ratings and the severity of anxiety in MDD (124). Moderate treadmill exercises prevent anxiety-like behaviors and the production of oxidative stress markers in the hippocampus, amygdala, and locus coeruleus (125). Importantly, antioxidants have the same effects as treadmill exercises, performing an anxiolytic effect (125), indicating that oxidative stress metabolites play an important role in mood disorders.

RAS overactivation is involved in oxidative stress *via* increasing Ang II levels and oxidative stress in the central nervous system is associated with depression (126) (**Figure 1B**). Ang II induces the production of superoxide anion and impairs cerebral microvascular endothelial function *in vivo* (127). Ang II stimulates inflammatory responses in the microvascular endothelium of the brain through AT1R, allowing more interaction between immune cells and the endodermis, and in turn, leading to disrupted BBB permeability partly *via* oxidative stress cascades (128). Ang II increases leukocyte adhesion 2.6-fold and BBB permeability 3.8-fold in male mice *via* oxidative stress-mediated cerebral microvascular inflammation (128). Furthermore, Ang II directly increases the production of ROS and subsequently induces lipid peroxidation and modification (129).

After systemic inflammation in the brain, nicotinamide adenine dinucleotide phosphate (NADPH) oxidase and inducible nitric oxide synthase (iNOS) activities rise, resulting in an accumulation of ROS and NO (126). During inflammation, Ang II promotes the oxidative stress process by increasing NADPH oxidase and iNOS activity in the BBB and PVN (126), and ARBs attenuate iNOS activity (126)(**Table 4**). Moreover, pretreatment with losartan at 3 mg/kg attenuates NO metabolite accumulation in hippocampal and cortical tissues (116). Treatment with telmisartan attenuates CIH-induced neuronal apoptosis in the hippocampus by suppressing NOS activity and inhibiting excessive NO generation (103). In addition, RAS blockers have a positive effect on depression as a comorbidity. Administration of RAS blockers prevents indices of systemic oxidative/nitrosative stress increased in rats with diabetes mellitus by inhibiting oxidative stress (131). Treatment with perindopril reduces severe acute respiratory syndrome-related coronavirus 2 spike protein-induced inflammatory and oxidative stress responses in cells and significantly blunted apoptosis and ROS (130).

**Table 4:** RAS blockers inhibit oxidative stress.

RAS Component	Compounds	Species	Mode of Administration	Measure Method	Main Findings	Reference
**Ang- (** 1–7)		Adult male Wistar rats			GPX↑ MDA↓	(37)
**AT_2_R**	Blocker PD-123177	Male Wistar rats	0.1 mg/kg/b.w. i.c.v. for 7 days	PA Y-maze	Antidepressants Memory↑ SOD↑ GPX↑ MDA↓	(37)
**ARB**	Losartan	Male Wistar rats	0.1 mg/kg i.c.v.		SOD↑ GPX↑ MDA↓	(59)
		Male Wistar rats	0.1 mg/kg/b.w. i.c.v. for 7 days	PA Y-maze	Memory↑ SOD↑, GPX↑ MDA↓	(37)
		Male LACA mice of CRS	20 mg/kg for 30 days	FST	Antidepressant MDA↓ Nitrite↓	(112)
	Telmisartan	Primary rat cortical neurons	10 ng/ml		NOX-4 mRNA expression↓ NADPH, ROS↓	(102)
		Male SD rats	10 mg/kg for 8 weeks		iNOS, NO↓ MDA↓	(67)
	Candesartan	Male Wistar Hanover rats	1 mg/kg/d, s.c. for 3 days		nNOS/eNOS activity↓ iNOS↓	(109)
		Male SD rats	0.1 mg/kg Orally for 5 days		ROS↓ Nitrite↓	(107)
	Irbesartan	Swiss albino mice of UCMS	40 mg/kg i.p./p.o.	MFST TST OFT	Antidepressant CAT↑ MDA↓	(100)
**ACEI**	Ramipril	Male LACA mice of CRS	10,20mg/kg for 30 days	FST	Antidepressant MDA↓ Nitrite↓	(112)
	Captopril	Male Wistar rats	0.1 mg/kg/b.w. i.c.v. for 7 days	PA Y-maze	Memory↑ SOD, GPX↑ MDA↓	(37)
	Perindopril	Male SD rats	1 μM Orally for 5 days		ROS↓	(107)
		THP-1 cells	100 µM		TNF-α, IL-17↓ Apoptosis↓ ROS↓	(130)

GPX, glutathione peroxidase; MDA, malondialdehyde; SOD, superoxide dismutase; MBT, Marble burying task; NOX-4, NADPH oxidase-4; NOS, Nitric oxide synthase; NO, nitric oxide; CIH, chronic intermittent hypoxia; iNOS, inducible nitric oxide synthase; CAT, catalase; HCD, high cholesterol diet; TABRS, thiobarbituric acid reactive substances, CUMS, chronic unpredictable mild stress; BDNF–TrkB-CREP, brain-derived neurotrophic factor-tropomyosin receptor kinase B–cAMP response element-binding protein; NSF, novel-suppressed feeding test, OFT, open-field test; TST, tail suspension test; FST, forced swimming test; SPT, sucrose preference test.

The limbic system, comprised primarily of the amygdala and hippocampus, includes widely distributed AT1 receptors and is sensitive to oxidative stress 184). Oxidative stress upregulates angiotensin-1 receptor levels and elevates NF-κB-mediated pro-inflammatory factors levels (IL-6, TNF-α) in these brain areas, leading to anxiety-like behaviors (132, 133). Candesartan significantly inhibits nuclear translocation of NF-κB expression, while also decreasing ROS levels and increasing IL-10 levels in the cortex and hippocampus (133). Captopril and losartan reverse the disruption of BBB permeability and prevent Ang II-induced enhancement of oxidative stress in the hippocampus (59, 134). Moreover, captopril and losartan decrease lipid peroxidation levels, reduce anxiety-like behaviors, and increase antioxidant enzymes including SOD and GPX (59, 134). Furthermore, antioxidant treatment improves Ang II-induced disruption of BBB permeability and prevented anxiety-like behaviors in rats (135). LPS injection raises the concentration of malondialdehyde (MDA), a marker of lipid peroxidation, in hippocampus tissue (99). Losartan prominently increases the activity of antioxidant enzymes and reduces lipid peroxidation, such as MDA (99, 136). Pretreatment with losartan (3 mg/kg) significantly decreases MDA levels and reverses the negative effects of LPS on the activity of CAT and SOD in the hippocampal and cortical tissues (116). Consistent with losartan, telmisartan suppresses CIH-induced lipid peroxidation and overexpression of inflammatory mediators in the hippocampus (103). Irbesartan, alone or in combination with fluoxetine, significantly decreases the levels of thiobarbituric acid reactive substances, CAT, and MDA, and reverses the reduction in GSH levels in unpredictable chronic mild stress (UCMS) mice (100). Taken together, RAS blockers improve emotional disorders through anti-oxidative stress effects.

### 4.5 RAS blockers inhibit stress responses

#### 4.5.1 RAS, corticotropin-releasing factor, and stress

In response to stress stimulation, the subgenual prefrontal cortex is suppressed and the amygdala is activated, which activates the HPA axis (137). As the major stress mediator and crucial regulatory center of the neuroendocrine system, HPA releases corticotropin-releasing factor (CRF) to regulate stress responses (138). When the HPA axis is activated, CRF is released from the paraventricular nucleus(PVN) into the portal circulation, stimulating the pituitary to produce and release adrenocorticotropic hormone (ACTH). ACTH further stimulates the glucocorticoid (GC) hormone cortisol synthesis and release by the adrenal cortex (139). Interestingly, increased CRF in the cerebrospinal fluid and hyperactivation of the HPA axis have been reported in both anxiety and depression (140, 141).

Corticotrophin-releasing factor or hormone (CRH) is a 41 amino acid neuropeptide that mediated the neuroendocrine, immunological, autonomic, and behavioral responses to stress (142). In various animal models of anxiety disorders, centrally injected CRF induces anxiety-like responses such as sleep disturbances, loss of appetite, and anhedonia (143). CRF antagonists show anxiolytic effects in a variety of animal models (143). In addition, a large literature indicates that stress responses upregulate the transcription and expression of Ang II-related receptors and enhance the expression of central Ang II (144–146). Ang II, as a stress hormone, increases the expression of CRF mRNA through AT1R, which contributes to increasing the expression of CRF receptors and promoting CRF release during stress (147, 148). Ang II stimulates ACTH and GC secretion through pituitary AT1R and/or activates Ang II afferent terminals innervating present in PVN neurons enhancing the stimulatory effect of CRF (149, 150)(**Figure 1C**).

The various stressors enhance Ang II levels and affect the expression of receptors. Pavel reported that ARBs prevent the response of the HPA axis to isolate stress and reduce the expression of CRH receptors and benzodiazepine receptors, demonstrating that ARBs exert powerful anti-anxiety properties by downregulating CRH receptor type and benzodiazepine receptors in stress models (151, 152). Saavedra et al. pretreated rats for 13 days with candesartan (0.5 mg/kg/day) followed by 24 h of isolation in metabolic cages, and they found that candesartan blocks the stress-induced augments of CRF in the cortical and prevents benzodiazepine receptors from binding in the paraventricular nucleus and cortex (153). Injecting Ang II-induced depressive-like behaviors *via* microglia activation and activates the HPA axis (28), pretreatment with candesartan (1.0mg/kg/days for 14 days) attenuates the response of the HPA axis to stress and reduces cold restraint stress (placed in plastic restraining devices and maintained at 4°C for 2 h)-induced ulceration of the gastric mucosa eventrally (154). Candesartan increases gastric blood flow by 40-50% and prevents gastric ulcer formation by 70-80% after cold-restraint stress (154). Administration of losartan effectively attenuates the stress-induced fear memory impairment and prevents the development of depression-like behaviors caused by chronic mild unpredictable stress (CMS) (116, 155). Low doses of candesartan completely reverse chronic restraint stress (2 h/21 days in tight plastic tubes)-induced memory deficits (156). Isolation stress increases AT1 receptor binding in the PVN and anterior pituitary. The administration of candesartan is sufficient to block the isolation stress (24h isolation in individual metabolic cages)-induced increased binding of AT_1_R to PVN and to reduce the HPA response to stress (157)(**Table 4**).

Systemic administration of angiotensin II receptor antagonist inhibits stress-induced anxiety (158). Ovariectomized rats treated with losartan decreased plasma corticosterone levels (*p*<0.05) and AT1R mRNA expression in the CA3 region of the hippocampus (159). Administration of losartan improves anxiety responses in stressed rats *via* blockade of the AT1 receptor within the amygdala under both non-stress and acutely stressed rats (160). In sub-chronic swim stress models, pretreatment with losartan (10 mg/kg) decreases anxiety-like and stress behaviors as manifested by enhancing the tendency to spend more time in the center area in the OFT (43). Chronic stress lead to neuropsychiatric disorders, such as anxiety and depression, the neuroprotective effect of losartan alleviates chronic fatiguing stress-induced anxiety-like behaviors (161). Moreover, pretreatment of losartan reveres the chronic restraint stress-induced increased anxiety-like behaviors and decreased motor activity (162).

Empirical studies in animals show RAS blockers exert antidepressive effects. Treatment with losartan significantly abolishes the increased Ang II level and prevents the development of stress-induced depression-like behaviors in UCMS rats (163). Chronic administration of irbesartan significantly increases swimming and climbing times, decreases immobility times in the MFST, and decreases immobility time in the tail suspension test in UCMS rats (100). Administration with telmisartan for five weeks notably prevents the depression-like behaviors in OFT and sucrose preference test (SPT) in animals under chronic stress (164).

#### 4.5.2 RAS, 5-HT, and stress

Serotonin (5-HT) is an important neuromodulatory transmitter and decreased 5-HT production may result in mood disturbance, aggression, and other neuropsychological impairment (165). There is an interaction between Ang II and 5-HT, particularly in the hippocampus, and brain Ang II regulates stress-related effects by modulating 5-HT release and synthesis (166, 167). The major serotonin metabolite 5-hydroxyindoleaceticacid (5-HIAA) is significantly elevated in the striatum after Ang II administration, indicating that Ang II increases the 5-HT levels (167). Furthermore, the AT1 receptor antagonist losartan reduces basal levels of 5HIAA (167). However, irbesartan (an ARB) increases time spent in the center of the OFT and elevates the 5-HT levels in UCMS rats (100), suggesting the biphasic response of Ang II on 5-HT synthesis (166). It is speculated that the biphasic response was related to the concentration of Ang II. Ang II stimulated tryptophan (TRP) hydroxylase at high concentrations to increase the synthesis of 5-HT. At low concentrations, an inhibitory effect is found, Ang II inhibits tryptophan hydroxylase resulting in decreasing 5-HT levels (166). Above all, it represents a subtle regulation of serotonin and the RAS system.

Perindopril (1.0 mg/kg/day) and candesartan (10 mg/kg/day) were administered *via* drinking water for 1 week, and serotonin levels increase in the prefrontal cortex and hippocampus, suggesting that decreased Ang II levels are associated with increased serotonin (168). Systemic administration of captopril increased serotonin levels and decrease depressive-like behaviors (115). In addition, captopril significantly increases the concentration of 5-HT and 5-HIAA in the parabrachial lateral nucleus (169). Administration with telmisartan for five weeks notably prevents the depression-like behaviors in OFT and SPT, and enhances expression of 5-HT transporter in the hippocampus of mice through activation of peroxisome proliferator-activated receptor δ, indicating RAS blockers improve stress-induced depressive symptoms in animals under chronic stress (164). Although these findings support that Ang II regulates stress by altering 5-HT levels in the brain, the association between Ang II and 5-HT in the formation of stress-related behaviors needs further investigation.

#### 4.5.3 RAS, sympathetic/parasympathetic, and stress

Stress generally triggers the autonomic nervous system, which is one of the major neural pathways (170). Depression is characterized by autonomic imbalance, with elevated sympathetic tone and weak parasympathetic tone, or both (171). A study of more than 600 subjects reports that depression and anxiety are shown to be more significantly and positively linked with the activation of the sympathetic nervous system (SNS), and depression is negatively correlated with parasympathetic nervous system (PNS) activation (172). SNS is stimulated in response to stress and increases the concentration of serum catecholamines to maintain body homeostasis (173). Indeed, patients with depression have been reported to have elevated catecholamines in plasma and cerebrospinal fluid (CSF) (174).

Norepinephrine (NE), one of the catecholamine hormones, is a major monoamine neurotransmitter that widely affects multiple brain regions (175). Noradrenergic hyperactivity has been shown to be an important component of the stress response and dysregulation of noradrenergic signaling has been implicated in the pathogenesis of anxiety and depression disorders. In fact, RAS has a complex bidirectional interaction with the autonomic nervous system activity under both physiological and pathophysiological conditions *via* receptors localized to peripheral and central sites of action (176). In the brain, Ang II increases sympathetic discharge and decreases vagal discharge. As a neuromodulator, Ang II stimulates ganglionic transmission and catecholamine release from adrenal medullary chromaffin cells and potentiated NE release from sympathetic nerve terminals in the periphery (177). In addition, there is some evidence that Ang II inhibits norepinephrine reuptake to promote neurotransmission (177). Administration of angiotensin-converting enzyme inhibitors can attenuate sympathetic neurotransmission and facilitate parasympathetic (175). In addition, Ang-(1–7) inhibits sympathetic tone and facilitates parasympathetic tone effects in experimental animal models, which will become an attractive treatment for autonomic nerve dysfunction (178).

In rat hypothalamic tissue, losartan partially reduces norepinephrine secretion (179). Also, noradrenaline levels are also shown to be significantly reduced in the striatum after chronic candesartan, although the mechanism is unclear (168). Ang II elevates catecholamines in the periphery and central, and pretreatment with Ang II receptor antagonism losartan significantly attenuates neuroendocrine responses, indicating Ang II activates sympathetic-adrenomedullary system activity through AT1R during stress (180). Although these findings support that Ang II regulated stress-related behaviors by altering NE levels, the association between Ang II and NE and sympathetic and parasympathetic in the formation of stress-related behaviors needs further investigation.

In clinical studies, elevated catecholamine activity impaired prefrontal cortex (PFC) function under stress and is associated with PTSD and other anxiety disorders (181). Chronic stress exposure leads to dendritic atrophy in PFC and enhances the noradrenergic NE system in the PFC (181). Cerebrospinal fluid norepinephrine concentrations are significantly higher in patients with chronic PTSD than in healthy subjects (182). Moreover, CSF norepinephrine concentrations are shown to be more significantly and positively link with the severity of PTSD symptoms, rather than plasma norepinephrine concentrations (182). Gold observed significant elevations in twenty-four-hour indices of norepinephrine secretion in both cerebrospinal fluid and plasma in severely depressed patients, compared with the control group (183). Clinical studies find that alpha-1 receptor blockers or alpha-2A receptor agonists can reduce the high concentration of NE release during stress, suggesting targeting the autonomic nervous system can be utilized as a pharmaceutical therapy for stress-related symptoms (181, 184). Taken together, the above finding indicates that RAS is involved in stress and that inhibiting RAS reduced the effects of stress.

### 4.6 RAS blockers elevate brain-derived neurotrophic factor levels

Brain-derived neurotrophic factor (BDNF) is a neurotrophic factor bound to its receptor tropomyosin-related receptor kinase B (TrkB). BDNF is associated with the neurobiology of depression and antidepressant effects (185). BDNF plays an important role in neuronal growth, maturation, and survival and it mainly has the following functions (186): (1) increase synaptic plasticity and affect learning and memory. (2) promote neurogenesis, especially in the hippocampus. Further, the expression of BDNF is regulated *via* cyclic adenosine monophosphate response element-binding protein (CREB), and the phosphorylated cyclic adenosine monophosphate response element-binding protein (pCREB) level in the hippocampus was one of the pathogenesis of depression (187). TrkB signaling is essential for antidepressants, activating TrkB and increasing levels of BDNF (188). Hippocampal biopsies showed that individuals with major depression reveal lower levels of BDNF and its receptor TrkB, and long-term use of antidepressants promotes increased BDNF levels (111). Chauhan et al. revealed that in depressed patients with low BDNF levels, after four weeks of antidepressant treatment, the serum BDNF levels and depressive symptoms significantly improve (189).

BDNF exhibits a negative regulatory effect on brain inflammation along with inflammation and oxidative stress (83, 189). RAS performs an integral effect in mediating BDNF, which is essential in the neurobiology of depression and antidepressant effects (34). Decreased BDNF levels in the hippocampal are associated with depressive-like behaviors in UCMS rats and losartan minimizes depressive-like behaviors *via* modulating the BDNF pathway (113, 190)(**Table 5**). Losartan treatment significantly elevates TrkB and p-CREB protein levels and reduces NF-κB protein, IL-6, and TNF-α mRNA levels, and facilitates the BDNF-TrkB-CREB, indicating that the TrkB signal promoted neuronal survival (111). Losartan exerts neuroprotective effects by alleviating neuroinflammatory responses and elevating BDNF levels in astrocyte (111). Oral administration of candesartan ameliorates chronic neuroinflammation-induced behavioral changes and apoptosis by inhibiting Ang II-induced NF-κB inflammatory signaling and enhancing the phosphorylated CREB and BDNF expression level in the cortex and hippocampus regions (133). In the case of AT1R blockade, AT2R activation increases the expression of AT2R mRNA (192). The antidepressant-like effect of losartan may increase the binding of Ang II to AT2R by inhibiting AT1R, finally, increase the surface levels of TrkB and coupling of TRK/FYN in the hippocampus and ventral medial prefrontal cortex (vmPFC) prelimbic aspects (34).

**Table 5:** RAS blockers reduce stress responses and elevate BDNF levels.

RAS Component	Compounds	Species	Mode of Administration	Measure Method	Main Findings	Reference
**AT1R**		AT1aR knockout mice in PVN		EPM	CRH gene transcription↓	(31)
**AT2R**	Agonist Novokinin	Male Wistar rats of T1DM	i.c.v.	EPM TMRA	Corticosterone↓	(32)
**ARB**	Telmisartan	Male Wistar rats of DM	0.5mg/kg, p.o. for 21days	FST OFT EPM	Antidepressant Serum cortisol↓	(101)
	Candesartan	Rats of CRS		EPM	Anxiolytic Sympathetic↓	(151)
		Male Wistar rats of isolation stress	0.5 mg/kg/day for 13 days		Anxiolytic CRF1R and BZ binding↓	(153)
		Male Wistar rats of isolation stress	4 mg/day per os for 3 months		Corticosterone↓ Aldosterone↓ Catecholamines↓ HPA response to stress↓	(157)
		Male Wistar rats of isolation stress	1 mg/kg/d, s.c. for 3 d		ACTH↓	(109)
	Losartan	Male SD rats of CMS	20 mg/kg/day for 7 weeks	SPT FST Y-maze test	Antidepressant Cognition↑	(163)
		Female long evans rats of Ovx	10mg/kg/day	SPT OFT EPM NORT	Anxiolytic Cognition↑ Corticosterone↓	(159)
		Male rats	2,4ug injected into amygdala	EPM	Anxiolytic	(160)
		Male LACA mice of CFS	10 and 20 mg/kg, ip for 21 days		Anxiolytic TNF-α, CRP↓	(161)
		Male Wistar rats of CFS	10 mg/kg for 10 days	OFT	Anxiolytic Stress↓	(162)
		Male LACA mice of CFS	20 mg/kg	FST	Antidepressant Corticosterone↓	(112)
		Wistar rats of DM	20 mg/kg for 2weeks	FST OFT	Antidepressant BDNF↑	(111)
	Valsartan	Male C57BL/6J mice of CUMS	40 mg/kg/d, p.o. for 4 weeks	NSF FST OFT TST SPT	Antidepressant Anxiolytic BDNF↑	(191)
**ACEI**	Ramipril	Male LACA mice of CFS	10,20mg/kg for 30 days	FST	Antidepressant Corticosterone↓	(112)
		Wistar rats of DM	10μg/bwkg/d for 2 weeks	FST OFT	Antidepressant BDNF↑	(113)
	Enalapril	Wistar rats of DM	40mg/bwkg/d for 2 weeks	FST OFT	Antidepressant BDNF↑	(113)

HPA, hypothalamic-pituitary-adrenal; CMS, chronic mild unpredictable stress; CFS, Chronic fatigue stress; CSR, chronically stressed rats; CRS, chronic resistance stress; BZR, Benzodiazepine receptors; CRH/CRF, Corticotropin-Releasing Hormone/factor; CRF1R, Corticotropin-Releasing Factor Receptor; UCMS, unpredictable chronic mild stress.

Reduced BDNF levels promote oxidative stress processes that lead to anxiety (120, 132). For example, social stress lowers BDNF and glutathione reductase levels, leading to oxidative stress-induced anxiety/depression-like behaviors in rats (193). Impaired hippocampal neurogenesis and reduced BDNF levels were observed in UCMS mice. Orally administration of valsartan (10–40 mg/kg/day, 4 weeks) promotes the hippocampus neurogenesis and the BDNF expression, exerting an antidepressant-like effect, which may be one of the mechanisms for its antidepressant (191). Importantly, the antidepressant-like property is dose-dependent, with the maximum effect obtained when valsartan is administered at a dose of 40 mg/kg/day (191).

## 5 Clinical data

To date, no randomized controlled trial has examined the impact of ACEIs or ARBs on depression. However, case reports and observational studies have demonstrated a bidirectional relationship between antihypertensive medications and depression, indicating mood-improving effects of RAS blockers, whereas other antihypertensive agents did not (**Table 6**). For example, losartan positively affects individuals with high-trait anxiety and prevents the development of anxiety disorders (205). In hypertensive individuals without a psychiatric history, discontinuation of valsartan (160 mg/day) is accompanied by anxiety symptoms such as palpitations, insomnia, and increased respiratory rate (206). These anxiety symptoms were significantly relieved with recontinuation of valsartan (80 mg/day) (206). A recent neuroimaging study showed that losartan prevents sustained amygdala activation in individuals with high-trait anxiety and leads to increased activation in other brain areas associated with threat processing, such as the insula and putamen (205). A recent study reports a significant association between the presence of an ACE inhibitor/ARB medication and decreased post-traumatic stress disorder symptoms compared with other blood pressure medications, including β-blockers, calcium channel blockers, and diuretics (207).

**Table 6:** Clinical data of RAS blockers.

Compounds	Study Design	Clinical Population	Main Findings	Reference
**SSRI** **SSRI+RAS**	PSM cohort study	SSRI users 30,311 SSRI+RAS 30,311 A total of 49,327 1997 to 2012	Risk for psychiatric hospital contacts↓	(194)
**ACEI** **ARB**	Nationwide population-based study in Danish	1,576,253 individuals 2005 to 2015	Rate of incident depression↓	(195)
**ACEI** **ARB**	Meta-analysis of Randomized clinical trials		Mental health domain of quality of life↑	(196)
**ACEI** **ARB**		378 patients with HBP	Rates of antidepressant usage↓	(197)
**ACEI** **CCB** **β-blockers**	Nationwide Population-Based Study in Danish	5.4 million individuals 2005 and 2015	Rates of depression↓	(198)
**ACEI** **ARB**	Prospective study	144,066 patients	Risk of mood disorders admissions↓ Risk for mood disorder↓ β-blockers and CCB higher risk	(199)
**Candesartan**	4 mg/d orally for 3 months	17 patients with T2DM	Interpersonal sensitivity↓ Depression ratings↓ Sensitivity of the adrenals to ACTH↓ Expression of AT1R↓	(148)
**Enalapril** **Captopril**	15.5 ± 1.54 mg/day for 1.66 ± 0.51 years	15 HBP patients	Anti-depressant Cognition↑	(200)
**Captopril**	12.5,50 mg	50-year-old woman with MDD	Anti-depressant	(201)
**Captopril**	12.5, 25 mg	41-year-old man with MDD	Anti-depressant	(202)
**Captopril**	50 mg t.i.d./4 weeks	Patiens with HBP	Anti-depressant	(203) (204)
**Losartan**	50 mg orally	30 anxious individuals	Anxiolytic	(205)

PSM, propensity scores matched; 95%-CI, 95%-confidence intervals; HRR, hazard rate ratio; HBP, hypertension; CCB, calcium antagonists; ACEI, angiotensin-converting enzyme inhibitors; ARB, angiotensin II receptor blockers; T2DM, type 2 diabetes.

Moreover, captopril and enalapril partly reverse the significant negative emotional effects of hypertension (12). Several cases have reported that captopril has mood-improving properties and antidepressant effects (202–204). Patients treated with captopril had significantly reduced total Hamilton Depression Scale (HAMD) scores and corrected neuroendocrine dysfunction during one-year follow-up (201). Moreover, captopril (12.5 mg b.i.d.2 weeks) decreases HAMD-21 scores and improves depressive symptomatology in patients with recurrent unipolar major depression (208). Captopril and enalapril improve depressive and anxiety symptoms in patients with hypertension (200). Hertzman reported that ten patients who suffered from hypertension and mood disorders (major depressive disorder or bipolar disorder) had improved mood with a combination of antidepressants and lisinopril, and no serious negative effects were reported by any of these patients while using lisinopril (209). A clinical trial reported that ACE inhibitors reduced the likelihood of depression risk and significantly improved general well-being, work performance, and cognitive function in 625 white men with mild hypertension administered captopril for 6 months (210). Two prospective multicenter randomized trials have shown that captopril has a significant tendency to reduce depressive symptoms compared with other antihypertensive drugs (211). Subsequently, another ACE inhibitor (enalapril) showed a significant improvement in health-related quality of life (HRQoL) compared to selective beta-blockers, although the overall tolerability of the two drugs was similar (212). In a clinical trial that enrolled 387 subjects aged 75+ years with hypertension in Italy, the results showed that the use of ACE inhibitors was associated with significantly better HRQoL among older adults (213). A case-control study enrolled 972 patients with both diabetes and depression, and the results suggest that those given ACEIs show a lower odds ratio for depression (OR 1.3, 95%confidence interval (95%CI):0.8–2.2) compared to beta-blockers (OR 2.6, 95% CI:1.1–7.0) and calcium channel blockers (OR 2.2, 95% CI:1.2–4.2) (214). Patients with type 2 diabetes and depressive symptoms received chronic candesartan administration for at least three months, which significantly improved interpersonal sensitivity and depression ratings and reset the HPA axis by reducing the sensitivity of the adrenals to ACTH and expression of AT1R (148).

In clinical and cohort studies, targeted RAS compounds have neuropsychiatric advantages through their anti-inflammatory properties, especially in depression (215). A Danish nationwide population-based study enrolled 1 576 253 subjects exposed to one of the six drugs(low-dose aspirin, statins, allopurinol, ACEIs, ARBs, and non-aspirin non-steroidal anti-inflammatory drugs) during the exposure period from 2005 to 2015. The incidence of depression decreased in patients taking ACEIs and ARBs, suggesting that these agents may act on inflammation and the stress response system (195). A recent meta-analysis identified 11 randomized controlled trials on the effects of antihypertensives on mental health and reported that ACEI and AT_1_R blockers have better effects on quality of life, anxiety, and mental well-being than placebo and other antihypertensives (196). Nasr analyzed data from 378 patients whom both suffered from hypertension and depression and found that patients being treated with an ACEI or ARB showed significantly lower rates of antidepressant usage, while beta-blocker and calcium channel blocker usage led to the highest rate of antidepressant usage (197). Consistent with these studies, Boal et al. analyzed cohort data and found that the use of RAS blockers in patients was associated with a low prevalence of depressive symptoms. The risk of affective disorders is reduced with the usage of ACEIs or Ang II receptor blockers, whereas the risk is increased with the administration of beta-blockers or calcium channel blockers (199). A nationwide population-based study showed that 3747190 subjects were exposed to antihypertensive drugs between 2005 to 2015. The findings of this study suggest that patients who continue to use classes of angiotensin agents, calcium antagonists, and β-blockers have significantly decreased rates of depression, whereas those using diuretic medication do not (198). A retrospective cohort study to investigate the distinct effects of antihypertensives on depression enrolled 181,709 patients and found that other antihypertensives may have a negative effect on the risk of depression compared with ARBs (216). Furthermore, 58.6 million patients aged 18–90 years were enrolled to study the influence of antihypertensive drugs on the onset and recurrence of psychiatric disorders (217). This study showed that ARB users exhibited the lowest incidence of psychotic, affective, and anxiety disorders for the first and recurrent diagnoses compared with CCB and β-blockers (217). However, there are limited randomized clinical trials on ACEI- and ARB-targeting medications and depression, which raises the possibility that future clinical research should conduct trials to examine these apparent advantages.

Despite the success demonstrated, a significant limitation is that not all research has revealed that ACEIs and ARBs have positive benefits on mood disorders, and these opposing results provide a new perspective for future clinical therapy. A small double-blind pilot study of eight patients was conducted to assess whether captopril has euphoric effects in the treatment of hypertension and found that the administration of captopril failed to elevate depressive mood (218). This result may be due to the small sample size of this study. A case report shows the dose of captopril is increased from 25 mg t.i.d. to 37.5 mg q.i.d. and the patient becomes intensely dysphoric within 2 days (203). When the captopril dose was raised to 37.5 mg q.i.d, anxiety and dysphoria reappeared. Furthermore, the dose was increased to 50 mg q.i.d., and the patient exhibited incoherent speech and suicidal ideation. It should be noted that although several studies have found that captopril has a positive effect on depression, its anxiolytic effect may be dose-dependent. In future studies, more attention should be paid to the frequency and different dosages of RAS blockers and the negative effects of different doses on mood.

In contrast, some epidemiological studies highlight concerns regarding possible links between the use of RAS blockers and increased risk of suicide, although the underlying mechanisms remain unknown (49). Mamdani et al. carried out a population-based nested case-control study, which included 964 patients and 3856 controls. The study found that ARB exposure is associated with a higher risk of suicide compared with ACEI (adjusted odds ratio, 1.63; 95% CI, 1.33–2.00). The preferred use of ACEI instead of ARB should be explored whenever possible, especially in individuals with severe mental disorders (219). Nonetheless, when a subsequent analysis was performed in 2020, a nationwide population-based propensity score matching study demonstrated that ARB use was not associated with an increased risk of suicide compared to non-ARB use (220). In summary, evidence from epidemiological studies suggests that the relationship between RAS medication use and suicide is inconsistent. Even if a direct cause-and-effect link exists, it is difficult to prove whether higher or lower RAS contributes to increased suicide risk.

Furthermore, most studies investigating the role of RAS in mood disorders have focused on pharmacological compounds that target ACE or AT1 receptors. Recently, several novel pharmacological compounds have been discovered, including ACE2 activators, Mas receptor agonists, AT2 receptor agonists, and renin blockers (221, 222), and further work to understand their roles is required.

## 6 Conclusion

A growing body of experimental and clinical data highlights the important role of the RAS in the pathophysiology of mood disorders. In this review, we presented an overview of the RAS, which consisted of two mutually antagonistic pathways that maintain balance through ACE2. The ACE/Ang II/AT1R classical pathway aggravates depression and anxiety by activating AT1R, while the non-classical pathway exerts anxiety/antidepressant effects by activating MasR. Moreover, RAS, mainly Ang II, is involved in the pathological process of depression by promoting inflammation, oxidative stress, and stress responses and reducing BDNF levels. Similarly, agents that inhibit RAS reduce inflammation, oxidative stress, and stress responses and facilitate neurogenesis. This may be the underlying mechanism of RAS blocker treatment for anxiety and depression. The full potential of RAS blockers as antidepressants and anti-anxiety drugs has not yet been elucidated. Hence, in future work, large-scale, randomized, controlled clinical trials are necessary to evaluate the therapeutic efficiency of RAS compounds in emotional disorders. RAS blockers need to be tested as potential therapies for emotional disorders, such as comorbid cardiovascular/cerebrovascular disease and depression. Furthermore, the role of RAS blockers in males and females in emotional disorders and pharmaceutical dosage in men and women should be carefully established.

Thus, RAS blockers may be a promising strategy for the treatment of mood disorders in the future. However, to realize the full therapeutic potential of RAS in mood disorders, further research is required.

## Author contributions

SG performed information collection and drafted the manuscript. FD supervised the review and approved the final version of the manuscript. All authors contributed to the article and approved the submitted version.
